# Establishment and Application of a Triplex Real-Time Reverse-Transcription Polymerase Chain Reaction Assay for Differentiation of PEDV, TGEV and PKV

**DOI:** 10.3390/vetsci11090413

**Published:** 2024-09-06

**Authors:** Jun Tu, Zhengdan Lin, Erchao Sun, Teng Yu, Weichao Zhang, Yumei Sun, Hechao Zhu, Pin Qian, Guofu Cheng

**Affiliations:** 1 College of Veterinary Medicine, Huazhong Agricultural University, Wuhan 430070, China; 2020302010132@webmail.hzau.edu.cn (J.T.);; 2 Guangxi Yangxiang Co., Ltd., Guigang 537100, China

**Keywords:** Porcine Epidemic Diarrhea Virus, Transmissible Gastroenteritis Virus, Porcine Kobuvirus, triplex RT-qPCR

## Abstract

**Simple Summary:**

This study reports the development of a triplex RT-qPCR assay for the simultaneous detection of Porcine Epidemic Diarrhea Virus (PEDV), Transmissible Gastroenteritis Virus (TGEV), and Porcine Kobuvirus (PKV) by targeting the conserved regions of their respective genes. The assay was optimized for specificity, sensitivity, and reproducibility, and demonstrated no cross-reactivity with other pathogens. It was validated against the Chinese standard single-assay method, exhibiting complete consistency in detecting these viruses in clinical samples. Finally, testing of 1502 clinical diarrhea samples revealed significant prevalence rates of PEDV, TGEV, and PKV, with mixed infections being common.

**Abstract:**

The pathogens responsible for porcine viral diarrhea are diverse, causing significant economic losses to the pig industry. PEDV and TGEV are well-known pathogens causing diarrheal diseases in pigs, leading to significant economic losses in the breeding industry. In contrast, the newly identified diarrhea virus, PKV, has not garnered as much attention. However, co-infection of PKV with PEDV results in more severe symptoms in piglets, such as acute gastroenteritis, and promotes increased replication of PEDV. Rapid and accurate diagnosis of viral diarrhea is essential for farms to identify pathogens early and mitigate economic losses. This study describes the development of a triplex real-time fluorescent quantitative RT-qPCR technique that can simultaneously detect three RNA viruses associated with porcine viral diarrhea: PEDV, TGEV, and PKV. To establish the triplex RT-qPCR method for the simultaneous detection and identification of the above three diarrhea viruses, conserved regions of the M gene of TGEV, the N gene of PEDV, and the 3D gene of PKV were selected to design specific primers and probes. After optimizing the reaction conditions, the method’s specificity, sensitivity, and reproducibility were evaluated. The triplex RT-qPCR method did not show a significant difference in PCR efficiency compared to the single RT-qPCR method. The method is specific to TGEV, PKV, and PEDV, exhibits no cross-reactivity with other pathogens, and demonstrates satisfactory sensitivity and reproducibility; the limit of detection (LOD) of PEDV, TGEV, and PKV is 11.42 copies/μL. Furthermore, the performance of the triplex RT-qPCR assay was compared with the Chinese standard single-assay method for detecting TGEV, PKV, and PEDV, showing complete consistency between the two methods (100% compliant). Subsequently, 1502 clinical diarrhea samples were collected from the Guangxi Zhuang Autonomous Region to investigate the local prevalence of TGEV, PKV, and PEDV and the positive rates were 16.38% (246/1502), 1.46% (22/1502), and 45.14% (678/1502), respectively. Co-infection of PEDV and PKV were most common, with a rate of 12.12% (182/1502). This study presents a valuable method for the rapid and simultaneous identification of PEDV, TGEV, and PKV in clinical animal farming practices, and provides a reassessment of the epidemiology of these diarrhea-causing viral pathogens in the Guangxi Zhuang Autonomous Region.

## 1. Introduction

Viral diarrhea in piglets leads to high mortality rates, causing significant economic losses to the swine industry [1]. In large-scale, intensive pig farming, viral diarrhea diseases are among the most common viral infections worldwide [2]. Porcine Epidemic Diarrhea Virus (PEDV), a coronavirus, primarily infects pigs, causing watery diarrhea and vomiting. The mortality rate among newborn piglets is exceptionally high, and the virus is highly infectious [3]. Transmissible Gastroenteritis Virus (TGEV) is an unsegmented, single-stranded positive-sense RNA virus; since the first reported cases of TGEV infection in pigs in 1933, outbreaks have been reported in many countries, including regions in southwest and central China [4]. The virus spreads through direct contact, causing vomiting, severe diarrhea, and dehydration in infected pigs, while newborn piglets exhibit acute and often fatal diarrhea [5,6].

In recent years, several novel viruses associated with diarrhea have been discovered; among these, PKV is a non-enveloped, single-stranded positive-sense RNA virus belonging to the family Picornaviridae. Since its initial discovery in diarrheic pig feces in China in 2007, PKV has been increasingly studied, revealing a widespread prevalence in both domestic pigs and wild boars. Infection rates vary among different age groups of pigs and are often associated with mixed infections of various pathogens. PKV infections also show typical seasonal patterns but lack regional specificity [7,8,9,10,11,12]. Recent studies have indicated that piglets co-infected with PEDV and PKV exhibit more severe clinical symptoms than those infected with PEDV alone, such as acute gastroenteritis and enhanced PEDV replication. Additionally, co-infected piglets show a significant reduction in the number of lymphocytes in the small intestine when compared to piglets infected with PEDV alone [13]. These findings underscore the need for the rapid diagnosis of PKV and co-infection with PEDV and TGEV.

PEDV, TGEV, and PKV belong to the family of enteric viruses. Clinically distinguishing between these viruses solely based on symptoms is challenging because all three can cause diarrhea and dehydration, presenting similar clinical manifestations. Infections typically manifest as single or mixed infections with other pathogens, complicating diagnosis [14]. A study in the Campania Region (Southern Italy) reported that over 65% of pigs tested seropositive for at least one porcine coronavirus, and more than half of the pigs in this region were also seropositive for Porcine Reproductive and Respiratory Syndrome Virus (PRRSV) [15]. Additionally, co-infections with pathogens such as PEDV and PKV [10], PEDV and TGEV [16], and the co-infection and evolution of swine enteric coronaviruses were also observed [17]. Rapid identification of pathogens is crucial due to potential mixed infections and the need for timely intervention [18,19]. Current diagnostic methods for PEDV, TGEV, and PKV include pathogen isolation, conventional or real-time RT-qPCR, enzyme-linked immunosorbent assay (ELISA), and other molecular techniques. However, these methods do not balance efficiency and detection speed simultaneously, failing to meet the urgent diagnostic needs during pig diarrhea outbreaks [20,21]. Reverse transcription real-time quantitative polymerase chain reaction (RT-qPCR) combines reverse transcription PCR and real-time quantitative PCR, and could detect multiple target nucleic acids in a single reaction using specific primers and probes. It enhances pathogen detection efficiency, conserves consumables and reagents, and reduces testing time and costs [22,23,24].

This study aimed to establish a triple RT-qPCR detection method for the simultaneous detection of PEDV, TGEV, and PKV. Specific primers and probes targeting the conserved regions of the M gene of TGEV, the N gene of PEDV, and the 3D gene of PKV were designed. This approach provides a rapid, accurate, and reliable detection technique for early clinical diagnosis, pathogen screening, and epidemiological investigation of these viruses in the region.

## 2. Materials and Methods

### 2.1. Primer and Probe Design

For the primer and probe design, representative sequences of the N gene of PEDV, the M gene of TGEV, and the 3D gene of PKV, as preserved in NCBI, were analyzed using MEGAX (v10.2) with several nucleotide sequences as shown in Figure S1. The optimal primers and probes were then designed and screened using Primer Premier 5 software (v5.0). For PEDV, the probe was labeled with FAM at the 5′ end and BHQ1 at the 3′ end. For TGEV, the probe was labeled with CY5 at the 5′ end and BHQ2 at the 3′ end. For PKV, the probe was labeled with VIC at the 5′ end and BHQ1 at the 3′ end. The primers and probes listed in Table 1 were synthesized by the Sangon Biotech (Shanghai, China) Co., Ltd. Primers and probes were adjusted to a 10 µM concentration using ddH_2_O.

### 2.2. Viral Strains and Standard Plasmid Construction

Standard plasmid pMD-PEDV-N, pMD-TGEV-M, and pMD-PKV-3D, containing the PEDV-N gene, TGEV-M gene, and PKV-3D 5′ non-coding region gene, respectively, were synthesized by the Sangon Biotech (Shanghai, China) Co., Ltd. (The synthesized sequences of standard plasmids are presented in Table S2). The plasmid served as the main research object to standardize, validate, and define the limits of detection in the experiments. The concentrations of the plasmids were adjusted to 1.142 × 10^9^ copies/µL using RNase-free ddH_2_O and mixed thoroughly before use. Copy numbers were calculated using the following formula:

$$copies/\mu L=\frac{(6.02\times 10^{23}\;copies/mol)\times (X\;ng/\mu g)\times 10^{-9}}{length\;\left(bp\right)\times 340\;g/mol}$$

### 2.3. Reagents and Instruments

A one-step RT-qPCR kit (FP314-01) was purchased from the TianGen Biotech Co., Ltd. (Beijing, China). A magnetic bead-based virus DNA/RNA extraction kit (NEMP0005-V-96) and an automated nucleic acid extraction instrument were purchased from the Harbin Genebase Biotech Co., Ltd. (Harbin, China). A biosafety cabinet was acquired from Haier Biomedical (Harbin, China). A real-time quantitative PCR instrument (BMS Mic-4) was obtained from the Shanghai Zhijiang Biotechnology Co., Ltd. (Shanghai, China). Additional equipment included a computer (DELL), a benchtop centrifuge, and pipettes (5 μL, 50 μL, 200 μL, 1000 μL) from Thermo Fisher Scientific Inc. (Waltham, MA, USA).

### 2.4. Sample Collection and Processing

From April 2023 to March 2024, a total of 1532 diarrhea samples were collected from multiple pig farms in the Guangxi Zhuang Autonomous Region (30 samples for methodological comparison and 1502 for clinical testing). The samples were processed using the magnetic bead-based virus DNA/RNA extraction kit, and nucleic acids were extracted using automated instruments and stored at −20 °C.

### 2.5. Optimization of RT-qPCR Amplification Conditions

Using the optimized concentrations of primers and probes as described above, standard plasmids were used as templates for RT-qPCR amplification. Specifically, 0.5 µL to 0.8 µL of upstream and downstream primers (10 µM each) and 0.5 µL to 0.8 µL of probe (10 µM) were added to a 20 µL reaction system. The TIANGEN^®^ FastKing One-Step RT-qPCR Kit (Probe) (TIANGEN, Beijing, China) was added to 10.8 µL, and nuclease-free water was added to bring the total volume to 20 µL. The annealing temperature optimization range was set from 56 °C to 60 °C to establish the optimal reaction system for triplex RT-qPCR. The amplification conditions were set as follows: 94 °C for 30 s, 94 °C for 5 s, annealing temperature for 30 s, for a total of 40 cycles. Real-time signals were detected at the end of each cycle, and the samples were stored at 25 °C for 300 s.

### 2.6. Construction of Standard Curves

Seven dilutions were selected from the plasmid standards and used as templates for RT-qPCR amplification reactions. The results were plotted with the logarithm of the copy number on the X-axis and the Ct value on the Y-axis to establish a standard curve and calculate the amplification efficiency using the formula: E = [10^−(1/slope)^ − 1] × 100%. At the same time, a standard curve for individual RT-qPCR amplifications was constructed in a manner consistent with the one described above, which was used to compare with the triplex RT-qPCR method we established. All the tests were repeated once.

### 2.7. Validation of Specificity, Sensitivity, and Stability

Total nucleic acids were extracted from a Foot-and-Mouth Disease Virus (FMDV) inactivated vaccine, a Porcine Parvovirus (PPV) vaccine, a Porcine Reproductive and Respiratory Syndrome Virus (PRRSV) live vaccine, a Porcine Circovirus Type 2 (PCV-2) inactivated vaccine, a Pseudorabies Virus (PRV) live vaccine, and a Gene-deleted live vaccine for infectious *Actinobacillus pleuropneumonia of swine* (*APP*), and a Classical swine fever virus (CSFV) live vaccine (all the vaccines used were purchased from commercial suppliers), and ddH_2_O using a total nucleic acid extraction kit according to the manufacturer’s instructions as a negative control. These nucleic acids were used as templates to validate the specificity of the developed triplex RT-qPCR method. A 10-fold serial dilution of standard plasmids (mixture of dilutions ranging from 1.142 × 10^6^ to 1.142 copies/μL) was tested using the established triplex RT-qPCR method to determine the limit of detection (LOD) (Ct value ≤ 35) and compare the amplification efficiency of single RT-qPCR and the triplex RT-qPCR method. To verify the stability of the method, separate tests were conducted to detect different concentrations of recombinant standard plasmids and compare the Ct values. Three different concentrations of positive samples were used as templates, and the triplex RT-qPCR method was performed on days 1, 7, and 30 (stored at −80℃), with each sample tested in triplicate. The within-group and between-group repeatability results were used to calculate the coefficient of variation for each result.

### 2.8. Comparison between Triplex RT-qPCR and PCR Methods of the Gold Standard in China

A total of 30 diarrheal samples from pig farms in the Guangxi Zhuang Autonomous Region were collected, and the detection results obtained from the triplex RT-qPCR method developed in this study were compared with PCR methods of the gold standard in China (according to national standards) to verify the accuracy of established detection methods. The detection method of PKV was built by the laboratory, with reference to Li Y et al. [25].

### 2.9. Clinical Application of Triplex RT-qPCR

The established triplex RT-qPCR detection method was applied to conduct an epidemiological study of PEDV, TGEV, and PKV in 1502 diarrheal pig samples (including small intestine tissues) collected from multiple pig farms in the Guangxi Zhuang Autonomous Region from April 2023 to March 2024. All samples were transported at 4 °C and stored long-term at −80 °C. Genomic information was extracted using a magnetic bead-based RNA extraction kit (NECVB, Harbin, China). A sample was considered positive if the Ct value was ≤35. Samples with a Ct value > 35 or without a real-time signal were deemed negative.

## 3. Results

### 3.1. Optimal Reaction Conditions

To determine the optimal amplification conditions for the triplex RT-qPCR method, a “matrix method” (Table S1) was used. This method involved varying the concentrations of upstream and downstream primers (0.5 μL, 0.6 μL, 0.7 μL, 0.8 μL) and probes (0.5 μL, 0.6 μL, 0.7 μL, 0.8 μL) specific to PEDV, PKV, and TGEV. The annealing temperatures were also optimized (56 °C, 57 °C, 58 °C, 59 °C, 60 °C). Figure S2 shows the effects of different annealing temperatures, probe concentrations, and primer concentrations on the real-time signal. The optimal real-time signal for FAM (indicating PEDV) was achieved at a probe concentration of 0.35 μM and a primer concentration of 0.25 μM (Figure S2A). Similarly, for CY5 (indicating TGEV) and VIC (indicating PKV), the optimal conditions were a probe concentration of 0.35 μM and a primer concentration of 0.25 μM (Figure S2B,C, respectively). The optimal reaction conditions, represented by sample A3, produced the lowest Ct values of 10.72, 11.60, and 11.75 for PEDV, TGEV, and PKV, respectively (Table S3). At an annealing temperature of 58 °C, the real-time signals for FAM, CY5, and VIC were most suitable (Figure S3C,H,M). Therefore, the selected optimal parameters were as follows: upstream and downstream primer concentrations at 0.25 μM each, and probe concentrations at 0.35 μM each. Among the five tested temperatures, 58 °C provided the best performance with the lowest Ct values of 13.00 for PEDV, 13.82 for TGEV, and 14.20 for PKV (Table S4).

### 3.2. Establishment of Standard Curves

**Figure 1:**
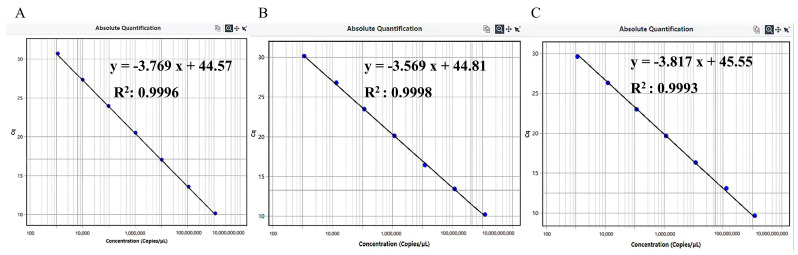
Standard curves of triplex RT-qPCR amplification (The same amplification system). (**A**) Standard curve of pMD-PEDV-N, y = −3.769x + 44.57, PCR efficiency: 84.22%, R^2^: 0.9996; (**B**) Standard curve of pMD-TGEV-M, y = −3.569x + 44.81, PCR efficiency: 90.61%, R^2^: 0.9998; (**C**) Standard curve of pMD-PKV-3D, y = −3.817x + 45.55, PCR efficiency: 82.80%, R^2^: 0.9993.

The standard curves for PEDV, TGEV, and PKV are shown in Figure 1. The slopes of the standard curves were −3.769, −3.569, and −3.817, while the PCR efficiencies were 84.22%, 90.61%, and 82.80% and the R^2^ were 0.9996, 0.9998, and 0.9993, respectively (Figure 1A–C). The R^2^ values and PCR efficiencies indicate a strong linear relationship between the template concentration and Ct value.

The PCR efficiencies for the single RT-qPCR standard curve of pMD-PEDV-N, pMD-TGEV-M, and pMD-PKV-3D, were 86.89%, 92.24%, and 84.30%, respectively (Figure S4). Compared to the single RT-qPCR reactions, the PCR efficiency of the triplex RT-qPCR method is only slightly lower and can be considered nearly comparable.

### 3.3. Validation of Specificity

**Figure 2:**
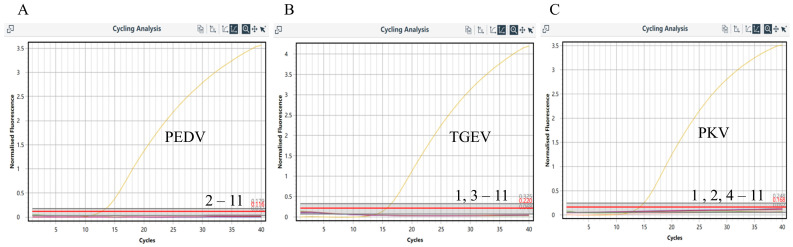
Results of the specificity experiment for triplex RT-qPCR. (**A**) PEDV amplification curve (only PEDV positive nucleic acid); (**B**) TGEV amplification curve (only TGEV positive nucleic acid); (**C**) PKV amplification curve (only PKV positive nucleic acid). 1: PEDV-positive nucleic acid; 2: PEDV-positive nucleic acid; 3: PKV-positive nucleic acid; 4: PRRSV-positive nucleic acid; 5: FMDV-positive nucleic acid; 6: APP-positive nucleic acid; 7: CSFV-positive nucleic acid; 8: PPV-positive nucleic acid; 9: PCV2-positive nucleic acid; 10: PRV-positive nucleic acid; 11: ddH_2_O-positive nucleic acid.

The specificity of the established method was validated using the triplex RT-qPCR method to detect the genomes of PEDV, TGEV, PDCoV, PTV, PRCV, PCV-2, PRV, CSFV, FMDV, PPV, and PRRSV. Total nucleic acid was extracted using the magnetic bead method, with negative controls included. The results showed specific amplification curves only for PEDV, TGEV, and PKV, while negative controls and other viral nucleic acids did not produce Ct values or specific amplification curves (Figure 2). This indicates that the established method possesses excellent specificity.

### 3.4. Validation of Sensitivity

**Figure 3:**
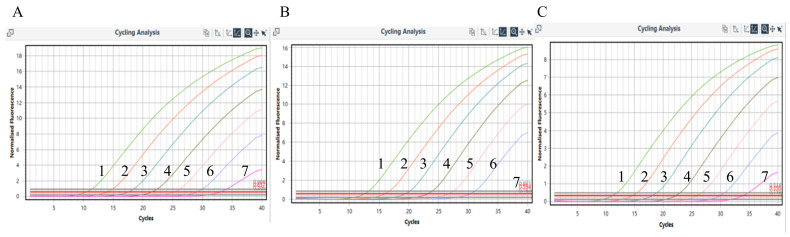
Sensitivity test results of triplex RT-qPCR. (**A**) PEDV Amplification Curve; (**B**) TGEV Amplification Curve; (**C**) PKV Amplification Curve. The concentrations of samples 1–7 in the graph are in the order of 1.142 × 10^6^–1.142.

The sensitivity test results (Figure 3 and Table 2) demonstrated that the LOD of PEDV, TGEV, and PKV is 11.42 copies/μL. This indicates good sensitivity for the method. The triplex RT-qPCR method yielded results that were nearly indistinguishable from those of the single RT-qPCR method in detecting the same sample (a range of dilutions). See Figure S4 for more details.

### 3.5. Validation of Stability

The stability of the developed method was confirmed, with within-group coefficient of variation values (CV) below 1.87% and between-group CV values below 1.03% (Table 3). These results demonstrate the method’s good stability.

### 3.6. Comparison with the PCR Methods of the Gold Standard in Clinical Sample Testing

To evaluate the accuracy of the triplex RT-qPCR, genomic nucleic acids were extracted from 30 clinical diarrheal samples and compared with the PCR methods of the gold standard in China. Both methods yielded the same detection results for PEDV, TGEV, and PKV, with detection rates of 23.33% (7/30), 6.67% (2/30), and 63.33% (19/30), respectively (Table 4). This indicates the method’s suitability for clinical detection.

### 3.7. Clinical Application of Triplex RT-qPCR

**Figure 4:**
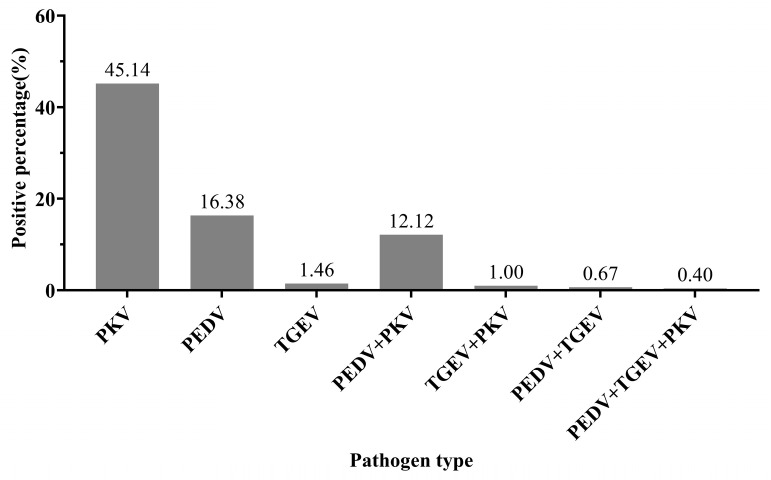
Results of triplex RT-qPCR detection for 1502 clinical samples.

The established triplex RT-qPCR method was applied to 1502 diarrheal samples collected from the Guangxi Zhuang Autonomous Region. The positivity rates for PEDV, TGEV, and PKV were 16.38% (246/1502), 1.46% (22/1502), and 45.14% (678/1502), respectively. Mixed infections were also observed, with PEDV + PKV being the most common at 12.12% (182/1502), followed by PEDV + TGEV at 0.67% (10/1502), PKV + TGEV at 1.00% (15/1502), and PEDV + TGEV + PKV at 0.40% (6/1502) (Figure 4).

## 4. Discussion

In recent years, the rapid development of large-scale pig farming, particularly during the autumn and winter seasons, has led to a significant increase in the incidence of viral diarrhea diseases [26]. Infections during the suckling and post-weaning stages of piglets severely affect their growth and development, resulting in diarrhea, emaciation, dehydration, and extensive mortality [27,28]. Infections during the production stage of sows result in both vertical and horizontal transmission within the pig population, characterized by recurrence and difficulty in eradication, ultimately leading to significant economic losses for farms [28]. Viral diarrhea diseases are widespread and harmful, posing substantial challenges for disease prevention and control in pig farms [29,30,31]. Among the common viruses causing pig diarrhea are TGEV, PEDV, PKV, Porcine Deltacoronavirus (PDCoV), Porcine Rotavirus (PoRV), Porcine Sapelovirus (PSV), Porcine Teschovirus (PTV), Porcine Bocavirus (PBoV), Porcine Torovirus (PDCoV), and Porcine Sapovirus (PSaV) [14,32,33]. Among these, PKV has a very high positivity rate and is a relatively new virus [7]. This study selected TGEV, PEDV, and PKV as research targets, necessitating the establishment of a diagnostic method that simultaneously detects these three pathogens.

In this study, we proposed a multi-target RT-qPCR detection method. This method is characterized by high specificity, sensitivity, and operational simplicity, and the reaction process does not require opening the tube lid, thus avoiding cross-contamination. The optimal primer concentration was crucial for specific target amplification, as both too high and too low probe concentrations could distort signals. To obtain the best signal intensity, we optimized the primer and probe concentrations using the “matrix method”. Ultimately, we obtained the optimal reaction conditions for triple RT-qPCR.

The detection results of the developed triplex RT-qPCR method were compared with those of the single real-time PCR method in China, showing consistency between the two methods. Subsequently, we tested 1502 clinical samples (rectal swabs and intestinal samples) to investigate the prevalence of PEDV, TGEV, and PKV in the Guangxi Zhuang Autonomous Region of China. Clinical sample analysis revealed positivity rates of 16.38% (246/1502) for PEDV, 1.46% (22/1502) for TGEV, and 45.14% (678/1502) for PKV. Mixed infections of PEDV and PKV were most common at 12.12% (182/1502), followed by PEDV and TGEV co-infections at 0.67% (10/1502), PKV and TGEV co-infections at 1.00% (15/1502), and PEDV, TGEV, and PKV co-infections at 0.40% (6/1502). In some pig farms, the PKV positivity rate was higher than that of PEDV and TGEV, indicating double or triple infections among PKV, TGEV, and PEDV. Ding et al. detected major pathogens such as PDCoV, PSaV, PEDV, and PRV-A from fecal samples collected across multiple provinces and cities. Co-infections were observed, including combinations like PDCoV + PSaV, PDCoV + PRV-A, PRV-A + PSaV, and PEDV + PDCoV, as well as triple infections such as PDCoV + PRV-A + PSaV and PEDV + PDCoV + PKV [14]. Similar results were obtained from fecal samples collected in southern provinces and cities in China, with the highest detection rate observed for PEDV at 52%. Co-infections of PEDV with PoRV, PDCoV, and other diarrhea-related viruses were commonly found [34]. The differences in positivity rates of PEDV, PoRV, and PKV in these studies could be attributed to factors such as the geographical distribution of samples, sample collection time, and season [35,36,37,38]. These co-infections are likely crucial factors contributing to the sustained occurrence of diarrhea.

The high prevalence of PKV infection and the frequent co-infection of PEDV and PKV underscore the necessity for simultaneous and rapid detection of multiple pathogens. When compared to other established assays for these three viruses [35,39,40,41], the triplex RT-qPCR method integrates the benefits of rapid diagnosis, cost reduction, and excellent performance in specificity, sensitivity, and stability, demonstrating its universal applicability through clinical sample testing.

Porcine intestinal viruses are complex, and besides well-characterized viruses like PEDV and TGEV, there are viruses with unclear characteristics, such as PKV. Importantly, some of these viruses are often detected in the same sample, indicating that mixed infections are common in some pig farms. Our research results indicated the presence of double or triple infections of PEDV, TGEV, and PKV in some pig farms in Guangxi, China. Double infections of pig intestinal viruses pose two major challenges to the prevention and control of viral pig diarrhea: they likely accelerate the evolution of individual viruses and the co-evolution of co-infected viruses, and they may lead to a recombination between co-infected viruses [17,42,43,44]. Rapid and accurate detection of pathogenic microorganisms is essential for controlling epidemics [45]. Accurate identification of pathogens and rapid differential diagnosis are crucial for epidemic control.

The triplex RT-qPCR detection method established in this study enables comprehensive and simultaneous detection of multiple pathogens. Compared to individual RT-qPCR or single real-time PCR, it significantly reduces costs and enhances operational efficiency, both of which have significant implications for the clinical prevention and treatment of these diseases.

## 5. Conclusions

The developed multiplex RT-qPCR method demonstrates high specificity and efficient detection capabilities, making it suitable for laboratory diagnosis and surveillance of TGEV, PKV, and PEDV. Most importantly, this method is well-suited for the differential diagnosis of clinical co-infections and facilitates early diagnosis. Due to its low cost, high efficiency, and ease of operation, neither the number of samples nor the type of test significantly impacts the time required to obtain results. This method allows for the rapid and simultaneous detection of three viral pathogens in diarrhea samples, providing a reliable diagnostic tool.

## Figures and Tables

**Table 1:** Primers and probes used in this study.

Name	Primer	Sequence (5′-3′) of Primer/Probe	Size (bp)
PEDV	PEDV-N-F	ATTCCCAAGGGCGAAAAT	96
	PEDV-N-R	ATCAACACCTTTTTCGACA	
	PEDV-N-P	FAM-TAGCAGCTTGCTTCGGACCC-BHQ1	
TGEV	TGEV-M-F	GTGCATTAGGAAGAAGCTATG	115
	TGEV-M-R	TTCATACCACCTGCAATTTTG	
	TGEV-M-P	Cy5-TTCCTCTCGAAGGTGTGCCAAC-BHQ2	
PKV	PKV-3D-F	TAGGAGACGAACGGGTGAC	124
	PKV-3D-R	TTGATGATGGAAGTGCCCA	
	PKV-3D-P	VIC-CTATATCGAAACCATACGCCACTCGC-BHQ1	

F, R, and P indicate forward primer, reverse primer, and probe, respectively.

**Table 2:** Sensitivity test results of triplex RT-qPCR amplification.

Plasmid Name	10-Fold Gradient Dilutions	10^6^	10^5^	10^4^	10^3^	10^2^	10^1^	1
pMD-PEDV-N	concentrations(copies/µL) Ct value	1.142 × 10^6^	1.142 × 10^5^	1.142 × 10^4^	1.142 × 10^3^	1.142 × 10^2^	1.142 × 10^1^	1.142
		10.70	14.16	17.74	21.66	25.43	29.36	33.24
pMD-TGEV-M	concentrations(copies/µL) Ct value	1.142 × 10^6^	1.142 × 10^5^	1.142 × 10^4^	1.142 × 10^3^	1.142 × 10^2^	1.142 × 10^1^	1.142
		12.57	16.06	19.42	23.23	26.75	30.38	N/A
pMD-PKV-3D	concentrations(copies/µL) Ct value	1.142 × 10^6^	1.142 × 10^5^	1.142 × 10^4^	1.142 × 10^3^	1.142 × 10^2^	1.142 × 10^1^	1.142
		11.29	14.18	18.26	22.32	26.11	30.11	34.10

**Table 3:** Verification results of detection reproducibility of triplex RT-qPCR method.

Pathogens	DNA/cDNA (Positive Samples)	Within-Group Test		Between-Group Test	
		Ct ^a^ (Mean ± S.D.)	C.V. ^b^	Ct ^a^ (Mean ± S.D.)	C.V. ^b^
PEDV	1	24.29 ± 0.45	1.86%	24.37 ± 0.06	0.26%
	2	27.95 ± 0.34	1.21%	28.27 ± 0.16	0.58%
	3	32.27 ± 0.51	1.58%	32.53 ± 0.25	0.76%
TGEV	1	24.91 ± 0.44	1.76%	25.09 ± 0.07	0.30%
	2	28.79 ± 0.28	0.96%	29.03 ± 0.18	0.60%
	3	32.96 ± 0.51	1.55%	33.23 ± 0.34	1.03%
PKV	1	24.12 ± 0.44	1.76%	25.05 ± 0.06	0.25%
	2	29.60 ± 0.31	1.05%	29.04 ± 0.14	0.48%
	3	32.96 ± 0.51	1.55%	33.33 ± 0.34	1.02%

^a^ Ct, cycle threshold; ^b^ C.V., co-efficient of variation.

**Table 4:** Results of triplex RT-qPCR method for clinical sample detection.

Samples	Types	PEDV		TGEV		PKV	
		tr-PCR ^b^	gb-PCR ^a^	tr-PCR ^b^	gb-PCR ^a^	tr-PCR ^b^	LDTs
1	fecal swab	-	+	-	-	+	+
2	fecal swab	-	-	-	-	+	+
3	fecal swab	-	-	-	-	-	-
4	fecal swab	-	-	-	-	+	+
5	fecal swab	-	-	-	-	+	+
6	fecal swab	-	-	-	-	-	-
7	fecal swab	+	+	+	+	+	+
8	fecal swab	-	-	-	-	+	+
9	fecal swab	-	-	-	-	+	+
10	fecal swab	-	-	-	-	-	-
11	fecal swab	-	-	-	-	+	+
12	fecal swab	-	-	-	-	+	+
13	fecal swab	-	-	-	-	+	+
14	fecal swab	-	-	+	+	-	-
15	fecal swab	+	+	-	-	+	+
16	fecal swab	-	-	-	-	+	+
17	fecal swab	-	-	-	-	+	+
18	fecal swab	-	-	-	-	-	-
19	fecal swab	-	-	-	-	+	+
20	fecal swab	+	+	-	-	+	+
21	fecal swab	-	-	-	-	-	-
22	fecal swab	-	-	-	-	-	-
23	fecal swab	+	+	-	-	+	+
24	fecal swab	-	-	-	-	-	-
25	fecal swab	-	-	-	-	+	+
26	intestinal tract	+	+	-	-	-	-
27	intestinal tract	-	-	-	-	+	+
28	intestinal tract	-	-	-	-	+	+
29	intestinal tract	+	+	-	-	-	-
30	intestinal tract	-	-	-	-	-	-

gb-PCR ^a^: PCR methods of the gold standard in China for the diagnosis of TGEV and PEDV (GB/T 36871-2018), respectively. LDTs: laboratory developed tests. tr-PCR ^b^: the triplex RT-qPCR method developed in this study. ‘’+’’ means positive. ‘’-’’ means negative.

## Data Availability

This manuscript includes all the detailed experimental procedures and results that should appear; if there are any problems with the manuscript, please contact us directly by email.

## Supplementary Materials

The following supporting information can be downloaded at: https://www.mdpi.com/article/10.3390/vetsci11090413/s1, Figure S1: Primer and probe design. The relevant sequences downloaded from the NCBI were used to design the primers and probes needed for detection by aligning the sequences to find the conserved sequences. The nucleic acid sequence for the PEDV-N gene was obtained from KJ399978. The nucleic acid sequence for the TGEV-M gene was derived from HQ462571. The nucleic acid sequence for the PKV-3D gene was obtained from JX164124; Figure S2: Optimization of fluorescence intensity. Influence of different probe concentrations and primer concentrations on the fluorescence signal of PEDV (A), TGEV (B), and PKV (C); Figure S3: Optimization of annealing temperature. Effects of different annealing temperature conditions on the fluorescence signals of PEDV (A, B, C, D, E), TGEV (F, G, H, I, J), and PKV (K, L, M, N, O); Figure S4: Standard curves of single RT-qPCR amplification (Single amplification system). (A) Standard curve of pMD-PEDV-N, y = −3.593x + 40.98, PCR efficiency: 86.89%, R2: 0.9997; (B) Standard curve of pMD-TGEV-M, y = −3.523x + 40.23, PCR efficiency: 92.24%, R2: 0.9993; (C) Standard curve of pMD-PKV-3D, y = −3.692x + 40.36, PCR efficiency: 84.30%, R2: 0.9993; Table S1: Optimization of primer and probe concentrations using the matrix method; Table S2: The nucleic acid sequences used in the study; Table S3: Optimization results of triplex RT-qPCR amplification conditions; Table S4: Exploration of annealing temperature in triplex RT-qPCR amplification reaction.
