# Supplementary material for: Establishment and Application of a Triplex Real-Time Reverse-Transcription Polymerase Chain Reaction Assay for Differentiation of PEDV, TGEV and PKV
Source: Vet Sci. 2024 Sep 6;11(9):413. doi: 10.3390/vetsci11090413 (PMC11435592; doi:10.3390/vetsci11090413)
Supplement: Supplementary file 1 [file vetsci-11-00413-s001.zip › vetsci-3141339-supplementary.pdf]

Supplementary Materials for

Establishment and Application of a Triplex Real-Time Reverse-Transcription Polymerase Chain Reaction Assay for Differentiation of PEDV, TGEV and PKV

Jun Tu [1,2], Zhengdan Lin [1], Erchao Sun [2], Teng Yu [2], Weichao Zhang [2], Yumei Sun[1], Hechao Zhu [2], Pin Qian [1] and Guofu Cheng [1*]

1 College of Veterinary Medicine, Huazhong Agricultural University, Wuhan 430070, China

2 Guangxi Yangxiang Co., Ltd., Guigang 537100, China

* Correspondence: chengguofu@mail.hzau.edu.cn

Figure S1

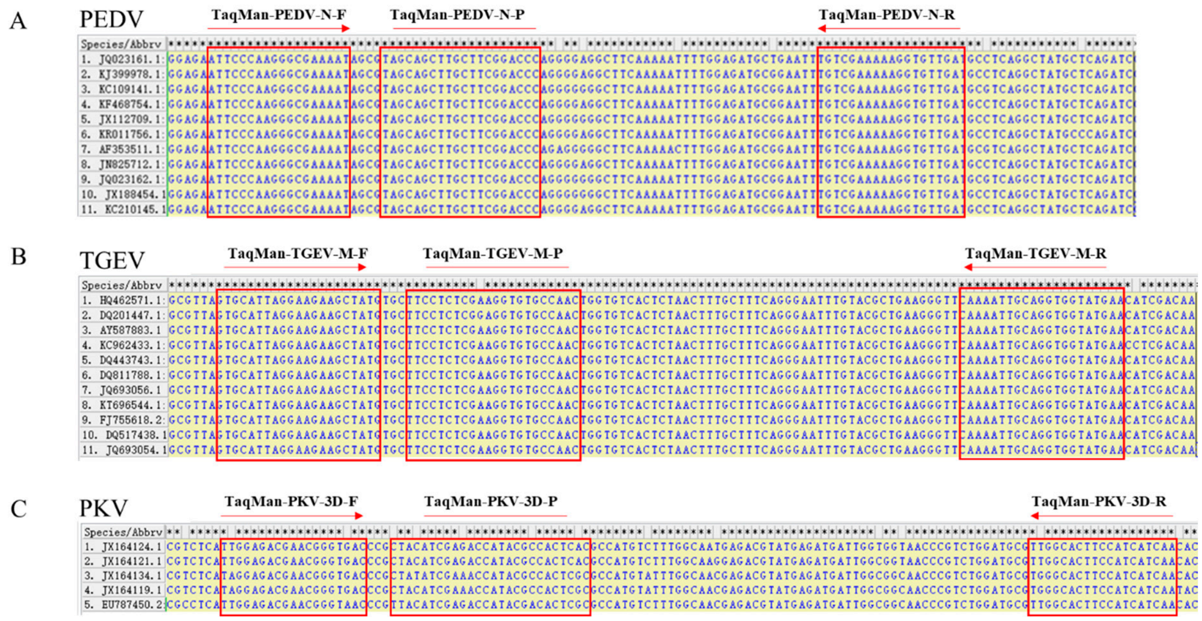

Figure S1. Primer and probe design. The relevant sequences downloaded from the NCBI were used to design the primers and probes needed for detection by aligning the sequences to find the conserved sequences. The nucleic acid sequence for the PEDV-N gene was obtained from KJ399978. The nucleic acid sequence for the TGEV-M gene was derived from HQ462571. The nucleic acid sequence for the PKV-3D gene was obtained from JX164124.

Figure S2

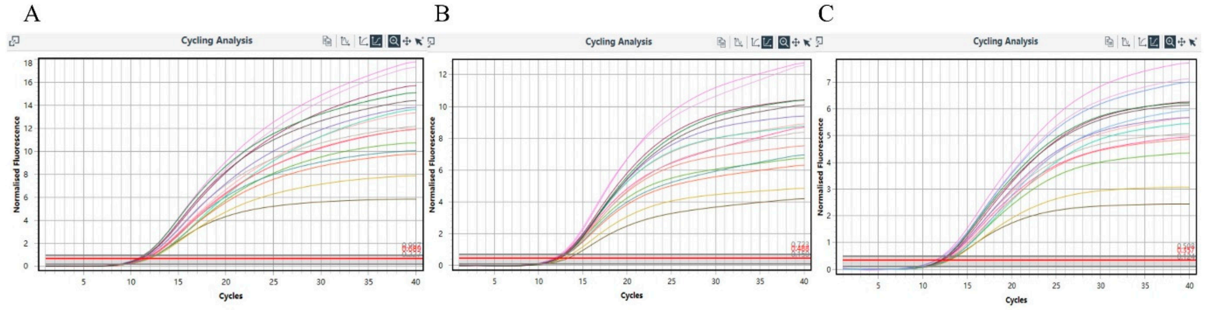

Figure S2. Optimization of fluorescence intensity. Influence of different probe concentrations and primer concentrations on the fluorescence signal of PEDV (**A**), TGEV (**B**), and PKV (**C**).

Figure S3

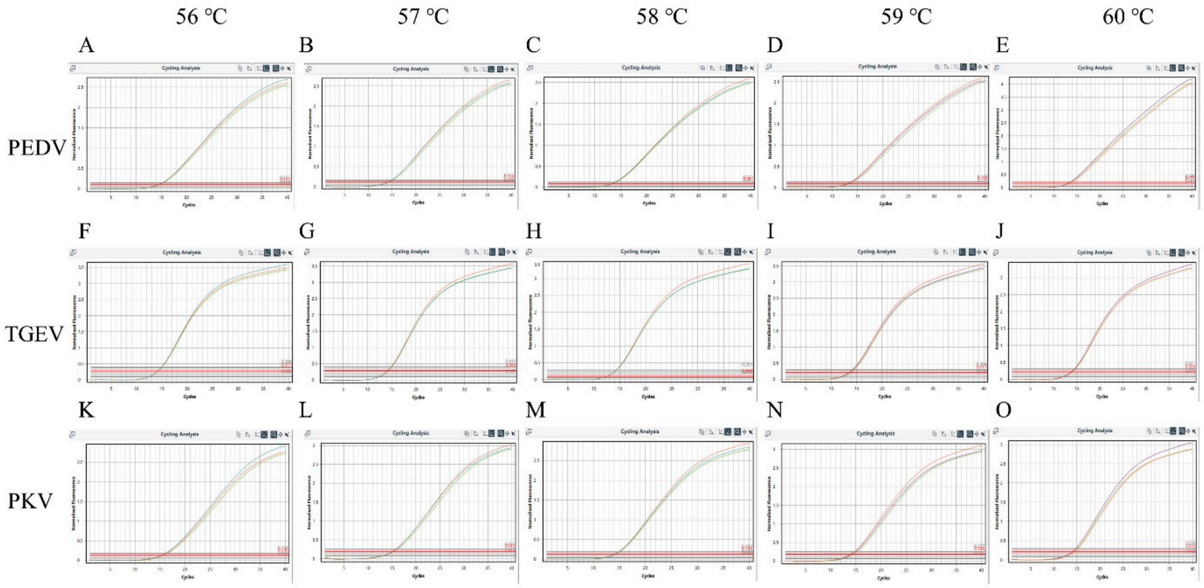

Figure S3. Optimization of annealing temperature. Effects of different annealing temperature conditions on the fluorescence signals of PEDV (**A**, **B**, **C**, **D**, **E**), TGEV (**F**, **G**, **H**, **I**, **J**), and PKV (**K**, **L**, **M**, **N**, **O**).

Figure S4

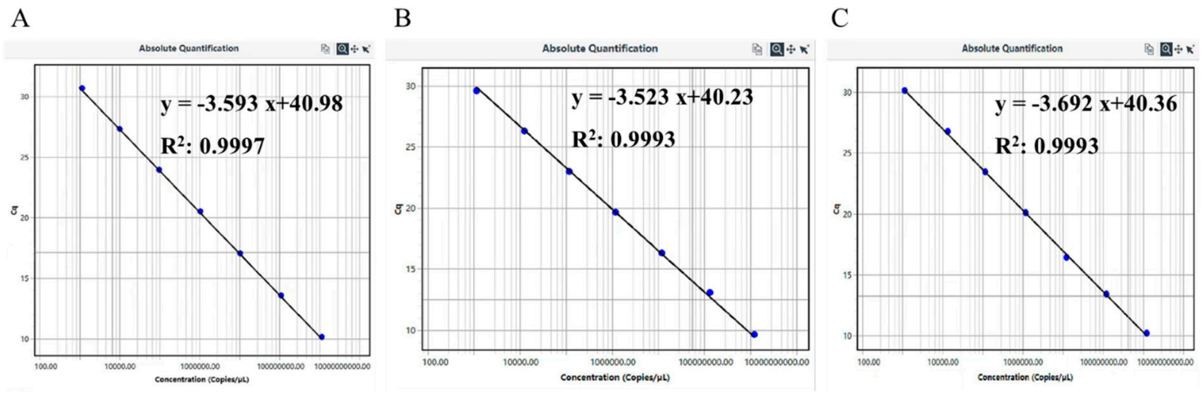

Figure S4. Standard curves of single RT-qPCR amplification (Single amplification system). (**A**) Standard curve of pMD-PEDV-N, y = -3.593 x+40.98, PCR efficiency: 86.89 %, R^2: 0.9997; (**B**) Standard curve of pMD-TGEV-M, y = -3.523 x+40.23, PCR efficiency: 92.24 %, R^2: 0.9993; (**C**) Standard curve of pMD-PKV-3D, y = -3.692 x+40.36, PCR efficiency: 84.30 %, R^2: 0.9993;

Table S1. Optimization of primer and probe concentrations using the matrix method

FPR	1	2	3	4
A	0.5/ 0.5/ 0.5	0.6/ 0.5/ 0.6	0.7/ 0.5/ 0.5	0.8/ 0.5/ 0.8
B	0.5/ 0.6/ 0.5	0.6/ 0.6/ 0.6	0.7/ 0.6/ 0.5	0.8/ 0.6/ 0.8
C	0.5/ 0.7/ 0.5	0.6/ 0.7/ 0.6	0.7/ 0.7/ 0.5	0.8/ 0.7/ 0.8
D	0.5/ 0.8/ 0.5	0.6/ 0.8/ 0.6	0.7/ 0.8/ 0.5	0.8/ 0.8/ 0.8

Table S2. The nucleic acid sequences used in the study.

Name	Sequence (5′ to 3′)
Nucleic acid sequences of PEDV-N (KJ399978)	atggcttctgtcagttttcaggatcgtggccgcaaacgggtgccattatccctctatgcccctcttagggttact<br>aatgacaaacccctttctaaggtacttgcaaataatgctgtacccactaataaaggaaataaggaccagcaa<br>attggatactggaatgagcaaattcgctggcgcatgcgccgtggtgagcgaattgaacaaccttccaattg<br>gcatttctactacctcggaacaggacctcacgccgacctccgctataggactcgtactgagggtgttttctgg<br>gttgctaaagaaggcgcaaagactgaacccactaacctgggtgtcagaaaggcgtctgaaaagccaatta<br>ttccaaatttctctcaacagcttcccagcgtagttgagattgttgaacctaacacacctcctacttcacgtgcaa<br>attcacgtagcaggagtcgtggtaatggcaacaacaggtccagatctccaagtaacaacagaggcaataa<br>ccagtcccgcggtaattcacagaatcgtggaaataaccagggtcgtggagcttctcagaacagaggaggc<br>aataataataacaataacaagtctcgtaaccagtccaagaacagaaaccagtcaaatgaccgtggtggcgt<br>aacatcacgcgatgatctggtggctgctgtcaaggatgcccttaaatctttgggtattggcgaagaccctga<br>caagcttaagcaacagcagaagcccaaacaggaaaggtctgacagcagcggcaaaaatacacctaaga<br>agaacaaatccagagccacttcgaaagaacgtgacctcaaagacatcccagagtggaggagaattccca<br>agggcgaaaatagcgtagcagcttgcttcggacccaggggaggcttcaaaaattttggagatgcggaatt<br>tgtcgaaaaaggtgttgatgcctcaggctatgctcagatcgccagtttagcaccaaatgttgcagcattgctc<br>tttggtggtaatgtggctgttcgtgagctagcggactcttacgagattacatataattataaaatgactgtgcc<br>aaagtctgatccaaatgtagagcttcttgtttcacaggtggatgcatttaaaactgggaatgcaaaacccca<br>gagaaagaaggaaaagaagaacaagcgtgaaaccacgcagcagctgaatgaagaggccatctacgat<br>gatgtgggtgtgccatctgatgtgactcatgccaatttggaatgggacacagctgttgatggtggtgacac<br>ggccgttgaaattatcaacgagatcttcgacacaggaaattaa
Nucleic acid sequences of TGEV-M (HQ462571)	atgaagattttgttaatattagcgtgtgtgattgcatgcgcatgtggagaacgctattgtgctatgaaatccg<br>atacagatttgtcatgtcgcaatagtacagcgtctgattgtgagtcatgcttcaacggaggcgatcttatttg<br>gcatcttgcaaactggaacttcagctggtctataatattgatcgtttttataactgtgctacaatatggaagacc<br>tcaattcagctggttcgtgtatggcattaaaatgcttataatgtggctattatggcccgttgttttggctcttacg<br>atttttaatgcatactcggaataccaagtgtccagatatgtaatgttcggctttagtattgcaggtgcaattgtt<br>acatttgtactctggattatgtattttgtaagatccattcagttgtacagaaggactaagtcttggtggtctttca<br>accctgaaactaaagcaattctttgcgttagtgcattaggaagaagctatgtgcttcctctcgaaggtgtgcc<br>aactggtgtcactctaactttgctttcagggaatttgtacgctgaagggttcaaaattgcaggtggtatgaac<br>atcgacaatttaccaaaatacgtaatggttgcattacctagcaggactattgtctacacacttgttggcaaga<br>agttgaaagcaagtagtgcgactggatgggcttactatgtaaaatctaaagctggtgattactcaacagag<br>gcaagaactgataatttgagtgagcaagaaaaattattacatatggtataa
Nucleic acid sequences of PKV-3D (JX164124)	cgtctcattggagacgaacgggtgacccgctacatcgagaccatacgccactcacgccatgtctttggcaat<br>gagacgtatgagatgattggtggtaacccgtctggatgcgttggcacttccatcatcaacaccatcatcaac<br>aacatttgtgtcctctctgctcttatccaacaccctgacttttcccctgaatccttcagaatattggcttatggtga<br>tgatgtgatttatgggtgtgatccacccatccatccatcattcatcaaggagttctacgacaaa

Table S3. Optimization results of triplex RT-qPCR amplification conditions

Sample number	Cycle threshold value		
	PEDV	TGEV	PKV
A1	11.98	12.23	12.68
A2	11.51	12.61	12.45
A3	10.72	11.60	11.75
A4	11.62	12.28	12.52
B1	11.36	11.73	11.81
B2	12.27	12.49	13.02
B3	12.14	13.00	13.12
B4	10.88	12.44	12.06
C1	11.57	11.82	11.92
C2	11.02	12.68	12.09
C3	11.73	12.23	12.55
C4	11.90	13.04	12.48
D1	11.20	13.03	12.20
D2	12.77	12.45	13.02
D3	11.14	12.17	12.22
D4	12.47	12.84	12.58

Table S4. Exploration of annealing temperature in triplex RT-qPCR amplification reaction

Plasmid Name	Annealing temperattre	56℃	57℃	58℃	59℃	60℃
pMD-PEDV-N	Mean Ct valte	14.22	14.05	13.00	13.27	13.26
pMD-TGEV-M	Mean Ct valte	14.29	14.29	13.82	13.59	13.95
pMD-PKV-3D	Mean Ct valte	15.59	14.60	14.20	14.27	14.26
